# Pilot Scale Production of Highly Efficacious and Stable Enterovirus 71 Vaccine Candidates

**DOI:** 10.1371/journal.pone.0034834

**Published:** 2012-04-17

**Authors:** Ai-Hsiang Chou, Chia-Chyi Liu, Cheng-Peng Chang, Meng-Shin Guo, Shih-Yang Hsieh, Wen-Hsueh Yang, Hsin-Ju Chao, Chien-Long Wu, Ju-Lan Huang, Min-Shi Lee, Alan Yung-Chi Hu, Sue-Chen Lin, Yu-Yun Huang, Mei-Hua Hu, Yen-Hung Chow, Jen-Ron Chiang, Jui-Yuan Chang, Pele Chong

**Affiliations:** 1 Vaccine R&D Center, National Institute of Infectious Diseases and Vaccinology, National Health Research Institutes, Zhunan, Taiwan; 2 Vaccine Center, Taiwan Centers for Diseases Control, Taipei, Taiwan; 3 Graduate Institute of Immunology, China Medical University, Taichung, Taiwan; The University of Hong Kong, China

## Abstract

**Background:**

Enterovirus 71 (EV71) has caused several epidemics of hand, foot and mouth diseases (HFMD) in Asia and now is being recognized as an important neurotropic virus. Effective medications and prophylactic vaccine against EV71 infection are urgently needed. Based on the success of inactivated poliovirus vaccine, a prototype chemically inactivated EV71 vaccine candidate has been developed and currently in human phase 1 clinical trial.

**Principal Finding:**

In this report, we present the development of a serum-free cell-based EV71 vaccine. The optimization at each step of the manufacturing process was investigated, characterized and quantified. In the up-stream process development, different commercially available cell culture media either containing serum or serum-free was screened for cell growth and virus yield using the roller-bottle technology. VP-SFM serum-free medium was selected based on the Vero cell growth profile and EV71 virus production. After the up-stream processes (virus harvest, diafiltration and concentration), a combination of gel-filtration liquid chromatography and/or sucrose-gradient ultracentrifugation down-stream purification processes were investigated at a pilot scale of 40 liters each. Although the combination of chromatography and sucrose-gradient ultracentrifugation produced extremely pure EV71 infectious virus particles, the overall yield of vaccine was 7–10% as determined by a VP2-based quantitative ELISA. Using chromatography as the downstream purification, the virus yield was 30–43%. To retain the integrity of virus neutralization epitopes and the stability of the vaccine product, the best virus inactivation was found to be 0.025% formalin-treatment at 37°C for 3 to 6 days. Furthermore, the formalin-inactivated virion vaccine candidate was found to be stable for >18 months at 4°C and a microgram of viral proteins formulated with alum adjuvant could induce strong virus-neutralizing antibody responses in mice, rats, rabbits, and non-human primates.

**Conclusion:**

These results provide valuable information supporting the current cell-based serum-free EV71 vaccine candidate going into human Phase I clinical trials.

## Introduction

Enterovirus 71 (EV71) infections have recently emerged in Asia as a serious cause of hand, foot and mouth disease (HFMD) in children that can lead to severe neurological complications and death [1]–[4]. Different types of vaccines against EV71 infection are being developed [4]–[6] and promising candidates are being evaluated in human Phase 1 clinical trials [6]. EV71 is a non-enveloped RNA virus of the family *Picornaviridae*, first identified in 1969 in the United States [7]. The EV71 virus particle contains a single molecule of plus sense ssRNA (7.5–8.5 kb). The complete nucleotide sequence of the EV71 prototype strain BrCr has been determined and contains four structural proteins: VP1, VP2, VP3 and VP4 [8], [9]. The cleavage of VP0 into VP2 and VP4 has been shown as a key capsid maturation step and also is important to viral RNA encapsidation [8], [10]. Two structural proteins VP1 and VP4 have been used for molecular epidemiological investigation and EV71 genotyping. Based on these studies, EV71 is currently classified into 3 genotypes, A, B and C and genotypes B and C are further divided into B1–B5 and C1–C5 sub-genotypes [11], [12]. Recent epidemics in Malaysia, Singapore, Taiwan, and Thailand were caused by B5 isolates; the virus strain that circulated in mainland China was C4 [12], [13]. Therefore, an effective EV71 vaccine should elicit strong cross-neutralizing antibody responses against different genotypes of EV71 in young children. Two different membrane proteins, human P-selectin glycoprotein ligand-1 (PSGL-1) [14] and human scavenger receptor class B, member 2 (SCARB2) [15] have been identified as cellular receptors for EV71. Transgenic mice carrying these genes are being engineered and should be useful for HFMD vaccine development.

The heat-inactivated EV71 virion produced from Vero cell grown in serum-containing culture media has been shown to elicit more effective immune responses than those obtained from the recombinant VP1 protein or DNA vector vaccines in mice [4]–[6], [16]–[18]. In the present study, we describe a scalable and reliable manufacturing process for a chemically-inactivated EV71 vaccine candidate that was capable of eliciting cross-neutralizing antibody responses in different animal immunogenicity studies. Our present findings provide valuable information for a serum-free, cell-based, heat-inactivated EV71 vaccine candidate going for human phase I clinical trials.

## Materials and Methods

### Ethics Statement

All experiments were conducted in accordance with NHRI Laboratory Animal Center guidelines, and approved by the NHRI Institutional Animal Care and Use Committee (Approval No. NHRI-IACUC-098033-A & NHRI-IACUC-099053-A).

### Animal welfare and steps taken to ameliorate suffering

There were five well-trained veterinarians in Animal Health Research Institute of Taiwan (AHRI) to take care of total 15 monkeys. Monkey immunogenicity study was performed according to study protocol NHRI-IACUC-099053-A. All juvenile monkeys (macaques) were kept in a secured specific pathogens free (SPF) room with locked double-doors and security camera to ensure no animal escape or irrelevant person entry. Individual monkey was housing in a double-door, squeeze-back cage (62×62×72 cm), with toys to enrich the environment. Monkeys can contact each other in vision but not physical. Monkeys were fed with fresh local fruits twice a day. Monkeys received the routine check up (X-ray, stool exam, and serological tests). The related record (critical blood chemistry (CBC) data, feeding record, and body weight) would be kept for 10 years. All monkeys were chemically restrained with Zoletil (1.4–4.5 mg/Kg) before immunization or bleeding. The vital sign was monitored by breath and heart beating. In addition, animal would be euthanasia and removed from the study if one of the following signs appears: (1) unable to eat or drink; (2) unable to relieve a severe pain even receiving a medical treatment by a certificated veterinarian; (3) a high-risk pathogen infection. Normally monkeys were immunized intramuscularly (leg) with the alum-adsorbed inactivated EV71 vaccine candidate; and they were boosted twice with the same dose three weeks interval after priming. Immunized monkeys were bled three weeks after the final boost, and 10 mL of blood was collected for detecting specific IgG antibody titer and neutralizing antibody titer. After blood samples collections, monkeys were placed back into original cages for recovery. Usually, 15∼20 minutes was needed and veterinarian monitored them. If an animal appeared to be in poor condition, an adequate medical treatment or environment improvement would be used to relieve the pain or distress. If the condition of an animal is out of control or is moribund, it may be euthanized by Pentobarbital (100 mg/Kg; iv injection) after a certificated veterinarian's review. After the study was completed, monkeys were still kept in AHRI with the same housing conditions. The health condition of monkeys will be checked every 3–6 months with CBC examination and body weight.

### Cells and virus

Master and working Vero cell and virus (E59 strain) seed banks were established following cGMP guidelines, characterized to fulfill the requirements for the manufacture of biological products by BioReliance (UK), and reported in our previous study [19].

### Medium selection in 75 cm^2^ T-flasks

The serum-containing (SC) medium contained basal medium DMEM purchased from Invitrogen (UK) and 5% fetal bovine serum (FBS) purchased from Moregate Biotech (Australia). Plus Vero and HyQ medium were purchased from Cesco Bioengineering Co. (Taiwan) and Hyclone (USA), respectively. VP-SFM and ExCell were supplied by Invitrogen (UK), and supplemented with 4 mM L-glutamine before use. Each 75 cm^2^ T-flask was inoculated with approximately 1.0×10^6^ cells and grown for 3 days in 20 mL medium. Every 24 hours, cell counts were determined by detaching cells in each T-flask using 4 mL of Trypsin-EDTA solution purchased from Invitrogen (UK). At each time point, cell counts were performed in triplicate.

### Pilot Scale purification of EV71 virus using liquid chromatography

EV71 virus stock was produced using the roller bottle technology, and purified by an AKTA Pilot liquid chromatography system purchased from GE Healthcare (USA) equipped with Sepharose Fast Flow 6 gel, and reported in our previous studies [10], [19].

### SDS-PAGE analysis and Western blotting

SDS-PAGE and Western blot analyses of the purified EV71 vaccine bulk were carried out according to previously-reported protocols [10].

### Animal immunogenicity studies

Mouse immunogenicity studies were conducted according to previously-reported protocols [10]. In parallel, rabbits, macaque monkeys and rats were immunized IM with 5–20 µg of protein formulated with 1.5 mg of alum per dose. Sera were collected two weeks after each immunization and used for immunological analysis.

### Virus neutralizing assay

Virus neutralization titer of each sample was determined using TCID_50_ assay according to the previously-reported protocols [10], [20].

### Enzyme-Linked Immunosorbent Assay (ELISA)

The VP2 epitope-specific quantitative ELISA was carried out according to previously-reported protocols [21]. The reactivity of the antibody to synthetic peptide VP1–43 [22] was analyzed by peptide-ELISA according to the protocol previously reported by Panezutti *et al.* [23].

### Stability profiles of EV71 vaccine

Eighty vials (3 mL each) containing either vaccine bulks (formalin-inactivated EV71 virion) or vaccine products (20 µg of protein of vaccine bulk formulated with 3 mg of aluminum phosphate in 1 mL of PBS) were stored at 4 or 25°C for stability studies. The stability of vaccine bulk was evaluated every 2 weeks for the first month and then every 3 months for up to 1 year using BCA protein assay, VP2 Q-ELISA, SDS-PAGE and Western blot analysis, and mouse immunogenicity study. The stability of the vaccine product was measured every month for the first 3 months and then every 3 months for 18 months using BCA protein assay (to analyze the unabsorbed protein), sterility tests and mouse immunogenicity studies.

### Residual DNA detection

Residual cellular DNA was detected by the Threshold® System (Model 0200–0500) using the Total DNA Assay Kit (MDC-R9004), both purchased from Molecular Devices Co. (USA). Five hundred mL aliquots of Zero Calibrator, Positive Control, High Calibrator from the Total DNA Assay Kit and the diluted samples (DNA content <400 pg/mL) were added into sterile DNA-free tubes. The double-stranded DNA in the tubes was denatured by heating at 105°C for 15 minutes, and the tubes were kept on ice until use. The standard solutions of single-stranded DNA into 0, 3.1, 6.3, 12.5, 25, 50, 100 and 200 pg per 0.5 mL were prepared using the High Calibrator. Labeling Reagent from the Total DNA Assay Kit was mixed with DNA samples and standards, and the mixtures were incubated at 37°C for 60 minutes. The filter block was pre-washed with the freshly-prepared substrate solution according to manufacturer instructions. Each assay mixture was transferred to the filter block, then filtered and washed. The concentration of DNA in the filter block was measured by Threshold® System and calculated using the included Threshold® software.

### Residual cellular protein detection

Vero cell protein-specific ELISA was used to measure the residual host protein content remained in EV71 vaccine samples. The in-house standard of Vero cell lysate was prepared from Vero cells grown in 200 mL SF medium in a roller bottle. The Vero cells were harvested and frozen at −80°C overnight. The cells were frozen and thawed several times to generate cell lysate standard. The total protein concentration of Vero cell lysate standard was determined by BCA protein assay. One hundred micrograms of Vero cell lysate was mixed with the complete Freund's adjuvant (CFA) and used to immunize rabbits 3 times for hyper-immune anti-Vero cell protein sera production. The quality and titer of rabbit anti-Vero cell protein sera were analyzed using Western blot. The Vero cell lysate standard was diluted into 0.15265, 0.3125, 0.625, 1.25, 2.5, 5 and 10 µg of protein/mL to generate the standard curve. One hundred mL of the diluted samples in the coating buffer were transferred into a 96-well microplate and tests were performed in duplicate. The microplate was sealed and incubated overnight at room temperature, washed with 0.05% Tween-20 in phosphate buffer saline (PBS), then blocked with 1% bovine serum albumin (BSA) in PBS at room temperature for 2 hours. Purified rabbit anti-Vero cell lysate IgG as prepared above was 1∶1000 diluted in 1% BSA/PBS, then added into each well and incubated at room temperature for 2 hours. The microplate was washed 3 times with 0.05% Tween-20 in PBS, a 1∶5000 dilution of goat anti-rabbit IgG conjugated to peroxidase (Chemicon USA) was added and incubated for 1 hour at room temperature. After PBS, the microplate was washed again 6 times with 1% BSA/PBS, then peroxidase substrate (KPL USA) was added and kept in the dark for 30 minutes. The stop solution (2N H_2_SO_4_) was added into the microplate, and the absorbance of each well was read at 450 nm by a microplate reader (Thermo Multiskan Spectrum USA) according to manufacturer instructions.

## Results and Discussion

### Evaluation and selection of serum-free medium

**Figure 1:**
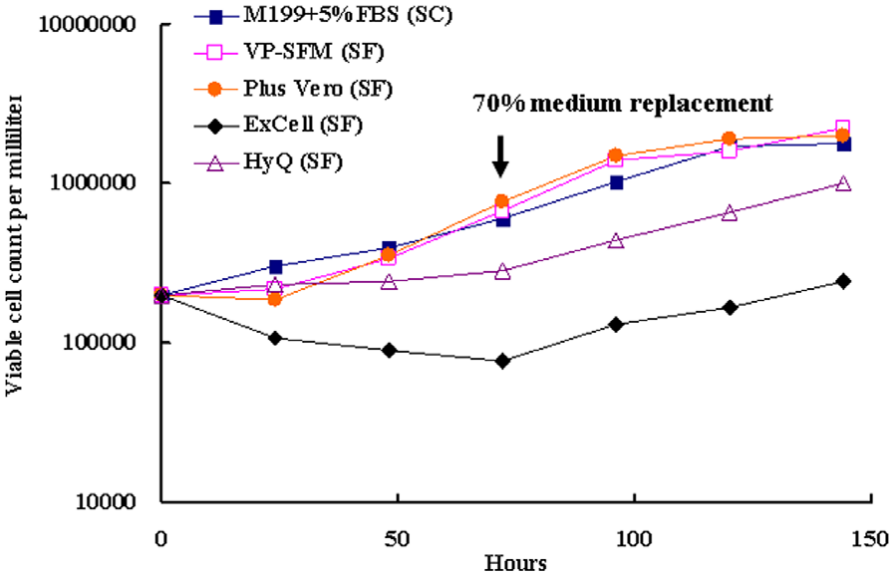
The growth profiles of Vero cell grown in serum-contain medium (SC) and different serum-free media (SF). Vero cells (2×10^5^ cell/ml) was cultured in 75T-flask and counted for viable cell number every 24 hours (0, 24, 48, 72, 96, 120 and 144 hours). The conditions for cell culture were described in the Materials and Methods section.

The performance of different media for Vero cell growth was screened and evaluated through the direct adaptation method [10], [17], [24]. The selection criteria were based on consistent cell growth performance over several passages. The cells were cultured in four different commercially-available SF media (Plus Vero, VP-SFM, HyQ and ExCell) and one SC medium (DMEM with 5% FBS supplement) in 75 cm^2^ flasks over three passages after inoculation. As shown in Figure 1, the average cell counts in the SC medium, VP-SFM and Plus Vero were found to be higher than those found in the Excell and HyQ SF media (p<0.05). We further examined the consistency of VP-SFM and Plus Vero SF media for Vero cell growth, and found three batches of VP-SFM to have similar levels in cell growth profile, whereas the culture in Plus Vero showed greater fluctuations and sometimes lower cell growth rates (data not shown).The current results suggested that the VP-SFM could consistently promote the cell growth and virus yield (see below). Thus, the VP-SFM was selected as the SF medium and was subsequently used in all later experiments.

### Optimization of EV71 virus yield

**Figure 2:**
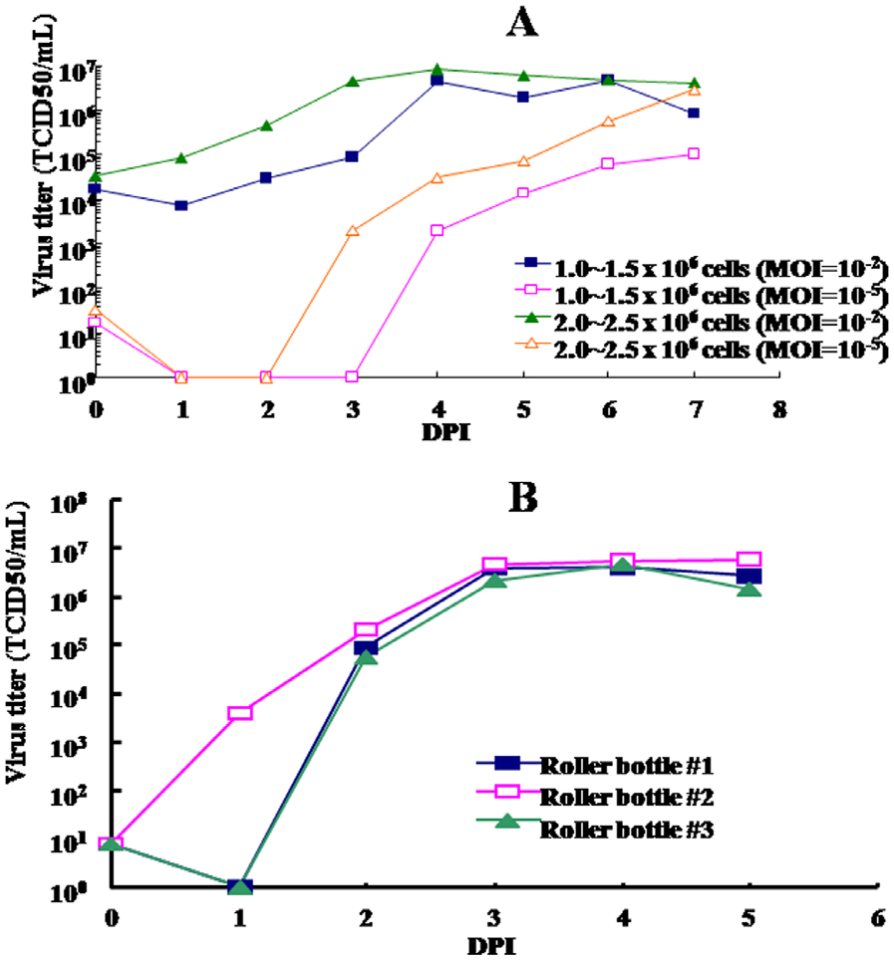
The optimization of the up-stream process for EV71 virus production. (A) The T-flask was seeded with either 1.0∼1.5×10^6^ or 2.0∼2.5×10^6^ Vero cells, then after 2–3 days the cells were infected by the different ratio of E59/EV71 virus. The effects of different M.O.I were detected in the kinetic profile of virus produced from Vero cell grown in the VP-SFM medium. (B) The consistency of 3 Lots of EV71 virus production in the roller bottles. Virus titer was detected every day by TCID50 for 5 days.

To evaluate the efficiency of E59/EV71 virus production in Vero cells grown in the VP-SFM medium, virus growth profile in T-flasks were done at four different multiplicities of infection (MOI): 0.01, 0.001, 0.0001 and 0.00001. As shown in Figure 2A, the virus titers in general decreased slightly during the first 2 days and then increased steadily after inoculation. The highest virus titer was 2×10^7^ TCID_50_/mL at 4 and 7 days post infection (DPI) for 0.01 and 0.00001 MOI, respectively. We also observed that increasing Vero cells (2–2.5×10^6^ cells) inoculation at the beginning of cell culture could generate a better virus yield (Figure 2A). To test whether temperature could influence virus growth and yield, the E59 strain was grown at various temperatures from 32 to 37°C. No significant differences in virus growth profile or yield were observed (data not shown). Therefore, 0.00001 MOI and 37°C were selected and subsequently used in all later production runs.

### Pilot scale up-stream process development

The virus growth profiles of three typical pilot scale production batches (200×200 mL roller bottles in each run) are shown in Figure 2B. In contrast to the results obtained from T-flasks, the virus titer was capable of reaching 10^7^ TCID_50_/mL at 3 DPI. The production kinetics of the E59 strain was similar when using 200 mL or 400 mL of culture medium (data not shown). EV71 virus was harvested and collected from the culture supernatant of each bottle at 5 DPI, prior to the onset of cytopathic effects. The potential cell debris was then removed by micro-filtration through a 0.65 µm membrane. To monitor and optimize the up-stream process, culture supernatants collected from each manufacturing run were assayed for their titers of infectious virus particles using the TCID_50_ assay and VP2 epitope contents by Q-ELISA as previously described [21]. The titers of infectious virus particles in the supernatants harvested from three cGMP lots were found to be 11.70, 6.31, and 10.5×10^6^ TCID_50_ units per mL for Lot #1, 2, and 3, respectively (Table 1). VP2 epitope contents were measured by Q-ELISA and found to be 11.9, 7.73, and 8.1 units of VP2 per mL in Lot #1, 2, and 3, respectively. The VP2 epitope contents were at least 3 folds higher than those obtained from virus produced in serum-containing medium [19]. The reason is unclear to us at this moment.

**Table 1:** Summary of in-process characterizations of EV71 vaccine bulk produced from serum-free medium.

Lot #	Process step	Total volume (L)	TCID_50_ a (×10^6^/mL)	Total Protein^b^ (µg/mL)	VP2 epitope^c^ (Unit/mL)	Recovery^d^ (%)	VP2 epitope/Total Protein (Unit/µg)
Lot #1	Harvest	41.0	11.7	1459.8+/−5.5	11.9+/−0.8	100	0.008
	Dif/Con^e^	1.10	38.9	825.6+/−9.5	531.0+/−14.5	119.7	0.64
	LC^f^	0.75	37.2	74.5+/−0.8	181.3+/−16.7	27.9	2.43
	Formalin inactivated	0.75		53.0+/−1.5	198.4+/−21.3	30.5	3.74
Lot #2	Harvest	40.0	6.31	1373.1+/−29.1	7.73+/−0.23	100	0.006
	Dif/Con	1.20	87.1	1351.7+/−13.6	249.0+/−12.47	96.6	0.18
	LC	0.88	31.4	64.4+/−0.89	130.4+/−9.07	37.1	2.02
	Formalin inactivated	0.80		50.3+/−1.27	130.4+/−6.7	33.9	2.59
Lot #3	Harvest	40.7	10.5	1318.7+/−27.6	8.1+/−0.31	100	0.006
	Dif/Con	1.00	223.9	1333.4+/−32.3	338+/−17.5	103.9	0.25
	LC	0.88	104.7	62.0+/−0.74	214.4+/−25.1	57.0	3.46
	Formalin inactivated	0.85		60.7+/−2.82	167.0+/−7.43	43.1	2.75

ND: not determined.

a TCID_50_ is the median tissue culture infective dose of EV71 that produces pathological cytopathic effects (CPE) in 50% of inoculated cell cultures.

b Total protein concentration was determined by the BCA protein assay.

c VP2-specific epitope content was determined by Q-ELISA [19].

d Recovery was calculated using the total VP2 epitope content at each step divided by the total VP2 epitope at the harvest stage.

e diafiltration/concentration step.

f gel-filtration chromatography purification.

The VP2 epitope content per 10^6^ TCID_50_ infective units were calculated to be 1.02 (11.9/11.7), 1.23 (7.73/6.31), and 0.77(8.1/10.5) for Lot #1, 2, and 3, respectively. These results are consistent with our previous small-scale research studies [21] that have shown the VP2 epitope content and the titer of infectious viral particles to be uncorrelated. In contrast, the ratio between VP2 content and total protein in the harvest shown in Table 1 was found to be relatively consistent in all three Lots (0.008, 0.006, and 0.006 for Lot #1, 2, and 3, respectively). Together, both results suggest that EV71 viral antigens are consistently produced from Vero cells grown in the VP-SFM culture system, but varying amounts of viral antigens are assembled to become infectious virus particles.

To facilitate downstream purification, the crude virus bulk was concentrated 20- to 40-fold using a 100 kDa cut-off diafiltration membrane in a tangential flow filter (TFF) cassette. As shown in Table 1, the TFF process was found to be efficient with high recoveries of viral antigens based on Q-ELISA units of VP2 (119%, 97%, and 104% for Lot #1, 2, and 3, respectively). The TFF process not only concentrated the viral antigens, but also removed significant amounts of cell proteins as the ratio between VP2 content and total protein as shown in Table 1 increased >40 fold (0.25/0.006 for Lot #3). However, Q-ELISA units of VP2 epitope per 10^6^ TCID_50_ infectious units were found to be 13.6 (531/38.9), 2.8 (249/87.1), and 1.5 (338/223) for Lots #1, 2, and 3, respectively. Again, the VP2 epitope content measured by Q-ELISA did not correlate well with TCID_50_ values. These results suggest that the un-assembled VP2 antigens were not removed from the virus concentrate at the 100 kDa TFF step. These results may also explain why 300 kDa cut-off diafiltration membranes used in the TFF step were less effective in the recovery of VP2 content as determined by Q-ELISA.

### Pilot scale downstream purification process

Three pilot-scale virus concentrates (∼1 liter) were purified using the AKTA Pilot liquid chromatography system (Table 1). The EV71 virus was generally identified and located in fractions 3 to 8 as determined by SDS-PAGE, Western blot, and TCID_50_. The EV71 virus fractions were pooled and concentrated using a 100 kDa TFF membrane. As shown in Table 1, ELISA units of VP2 epitope per 10^6^ TCID_50_ infectious units were found to be 4.87 (181.3/37.2), 4.15 (130.4/31.4), and 2.05 (214.4/104.7) for Lot #1, 2, and 3, respectively. These results suggest that the virus bulk pooled from multiple fractions may still contain various amounts of defective and infectious EV71 particles. As shown in Table 1, the ELISA units of VP2 epitope per µg of protein was found to be 2.43, 2.02, and 3.46 for Lots #1, 2, and 3, respectively. These results are close to those obtained from the 3 previously reported research Lots (3.8, 5.3 and 3.4) [21]. Furthermore, the overall recovery yield based on VP2 content determined by ELISA varied (27.9, 37.1, and 57.0% for Lots #1, 2, and 3, respectively) in these three pilot runs. The gel-filtration chromatography removed >95% (>1300 decreased to 62 µg/mL of total protein for Lot #3) of potential contaminants. The residual host cell proteins within the virus bulks was determined using Vero cell-specific ELISA as described in the Materials and Methods and found to be 2.72, 2.61, and 2.38 µg/mL for Lots #1, 2, and 3, respectively. The current results indicate that 5% (2.61/50.3 of Lot #2) of total protein are residual Vero cell proteins within the virus bulks. As a result, there is still room for improvement in the chromatographic purification process.

Two hundred mL of formalin-inactivated chromatographically-purified viral stock (64 µg/mL) was further purified using sucrose gradient ultracentrifugation as previously described [10]. Two kinds of EV71 virus particles were detected by TCID_50_ assay and Western blot analysis, the defective and infective particles were found in the fractions containing 25–28% and 35–38% sucrose, respectively (data not shown). When protein concentrations of both EV71 particle pooled fractions were measured by BCA method, it was found that there were 3 times more defective particles (52 µg/mL) than infectious particles (15 µg/mL). Like in our previous report [10], the purified infectious EV71 virus was shown to be consisted of all 4 structural proteins VP1, VP2, VP3 and VP4. In contrast, in the defective viral particles VP0, VP1 and VP3 protein bands were found to be the major components as shown in the SDS-PAGE and Western blot analysis [10]. Although ultra-pure EV71 virus bulk could be obtained by combining the purification process of liquid chromatography with sucrose gradient ultracentrifugation, the recovery yield was less than 20% of the chromatographically-purified viral stock. Since the defective particle fractions are also immunogenic and could elicit neutralizing antibody responses in both immunized mice and rabbits as previously reported [10], [19], [21], it was therefore decided that the pilot-scale downstream purification would use gel-filtration chromatography.

### Chemical inactivation optimization

**Figure 3:**
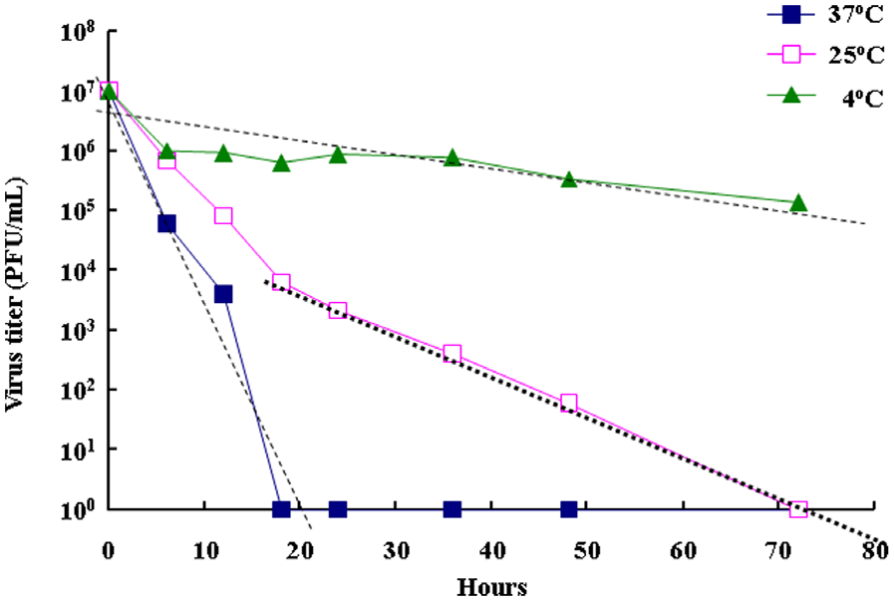
The temperature effect in the kinetic of formalin-inactivation of EV71 virus. Purified E59/EV71 was inactivated by 0.025% (v/v) formalin at different temperature and the residual of virus infectivity was detected by plaque assay performed triplicate at different time points.

In previous reports [10], [19], [21], [22], [25], the purified EV71 virus bulk could be inactivated with formalin either at 4°C for weeks or 37°C for a few days, but these inactivation processes had not been validated for virus reversion. Therefore, the formalin-inactivation kinetic was performed at 4, 25, and 37°C. The results are shown in Figure 3, no infectivity was detected in the samples taken 18 and 72 hours after exposure to 0.025% (v/v) of formalin at 37 and 25°C, respectively. After 24 days, no infectious EV71 was observed in the samples taken from 4°C. Based on the current inactivation kinetic, the complete viral inactivation for 10^12^ TCID_50_ titer virus bulk would require 2.16, 12.6 and 31.64 days at 37, 25, and 4°C, respectively (Figure 3). After 0.22 µm sterile filtration, the total protein concentration measured by the BCA assay was found to have 20–30% loss of protein content during the inactivation and sterile filtration steps (Table 1). However, the Q-ELISA unit in the vaccine bulk was found to be similar to the value observed in the virus bulk before inactivation. These results suggest that formalin inactivation did not modify the VP2 epitope content as determined by VP2 Q-ELISA. In addition, the residual DNA in the EV71 vaccine bulk was determined according to the protocol described in the Materials and Methods section and found to be 16.67, 8.13, and 2.87 pg/mL for Lot #1, 2, and 3, respectively. These levels would pass the acceptance criteria for human vaccine (10 ng/dose) based on FDA guidelines.

### Immunogenicity studies of EV71 vaccine candidates

**Figure 4:**
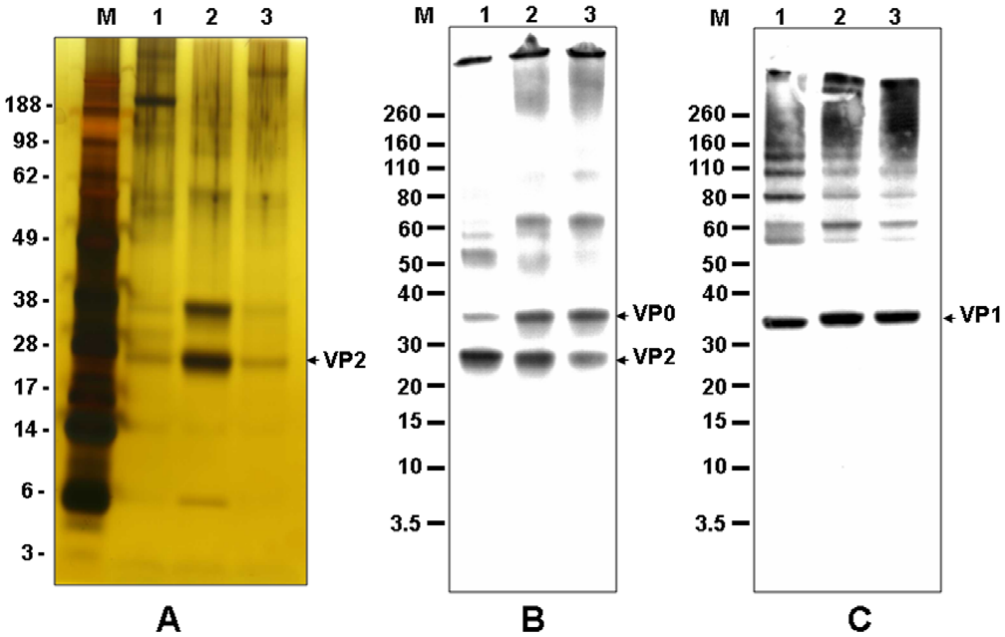
The stability profiles of different Lots of EV71 vaccine products stored at 4°C for various time and analyzed by SDS-PAGE (Panel A) and Western blot (Panel B & C). Lane M is MW Markers; lane 1 is EV71 vaccine product produced from serum-containing medium and stored at 4°C for 26 months; lanes 2 and 3 are EV71 vaccine products derived from Lot #1 and 2 and stored at 4°C for 13 and 4 months, respectively. Monoclonal antibody used in the Panels B and C are MAB979 specific for VP2 and N16 specific for VP1, respectively.

The quality of EV71 vaccine bulks (formalin-inactivated EV71 whole virions) was analyzed by SDS-PAGE and Western blots (Figure 4). After silver-staining SDS-PAGE gels, the dominant protein bands with molecular weight (MW) 28 and 38 kDa were observed in the vaccine bulks (Figure 4A, lanes 2 & 3). From our previous studies [10], [19], [21], [22], [25], protein bands with MW 38 and 28 kDa correspond to VP0/VP1 and VP2, respectively. Since the gel was loaded with the same amount of vaccine bulk based on protein concentration (2 µg per lane), it was suggested that Lot #1 contained more VP0/VP1 and VP2 antigens than Lot #2. This observation is consistent with the ratio of VP2 epitope/total protein found in Lots #1 (3.74) and #2 (2.59). The vaccine bulks were further analyzed by Western blot using two monoclonal antibodies (MAb) with different specificity: MAB979 recognizes an epitope of VP2, while N1 reacts with VP1 [21], [22], [25] As shown in Figures 4B and C, both MAb recognized similar antigen patterns in both Lots #1 and 2. We observed similar Western blot results from Lot #3 (data not shown). These results indicate that the antigen patterns of EV71 vaccine bulks were produced consistently throughout the current manufacturing process.

Mouse immunogenicity studies summarized in Table 2 revealed that the formalin-inactivated EV71 virions were highly potent in eliciting virus neutralization titer that were correlated with VP2 epitope units [10], [19], [21], [22], [25]. Although the neutralization titer was decreased (2- to 14-fold), sera from mice immunized twice with EV71 vaccine bulk were capable of cross-neutralizing other isolates from B4, B5 sub-genotype and C4 genotype (Table 2).

**Table 2:** Immunogenicity studies of EV71 vaccine bulks.

Animal Model	Vaccine Lot #	Total proteins (µg/dose)	VP2 Q-ELISA unit/dose	IgG Titer (GMT)	TCID_50_ Neutralization titer (GMT)			
					B4/E59	B4/S0302	C4	B5
BALB/c Mouse	1	2	7.5	4,525	443	256	32	128
	2	2	5.2	2,934	233	ND*	64	256
	3	2	5.5	2,851	234	ND	ND	ND
Rat	2	10	26	12,800	654	ND	64	128
Rabbit-G1	1	5	18.8	>32,000	13,573	5,677	8,192	5,677
Rabbit-G2	1	10	37.6	>32,000	13,318	ND	13,520	16,384
Rabbit-G3	2	5	13	>32,000	17,093	ND	ND	ND
Rabbit-G4	2	10	26	>32,000	19,308	ND	ND	ND
Macaque	1	10	37.6	>32,000	5,623	ND	5,623	3,169
Macaque	3	5	13.8	25,600	3,169	ND	ND	ND
Macaque	3	10	27.6	25,600	8,912	ND	ND	ND

Eight mice per group and 5 rats per group were immunized twice with formalin-inactivated EV71 virion formulated with alum. Two rabbits per group and individual macaques were immunized three times with formalin-inactivated EV71 virion formulated with alum. The immunization protocol, IgG tier and virus neutralization assay are described in the Materials and Methods section.

* not done.

The results of other animal (rats, rabbits and macaques) immunogenicity studies are shown in Table 2. Sera from the immunized animals were found to have strong cross-neutralization titer against other B subgenotypes and C genotypes of EV71. Our current results are consistent with the conclusions reported by both Bek *et al.* and Dong *et al*., [26], [27]. They had produced formalin-inactivated EV71 vaccine candidates based on C4 genotype virus that could elicit cross-genotypes neutralizing antibody responses in mouse and non-human primate models [26], [27]. They suggest that the common neutralization epitopes of EV71 virus are most likely conformational that could elicit strong cross-neutralizing antibody responses against different EV71 genotypes.

### Stability profiles of vaccine bulks and formulated vaccine candidates

The stability profile of 2 different lots of EV71 vaccine bulks based on protein assay and VP2 Q-ELISA are summarized in Table 3. After 1 year of storage at either 4 or 25°C, there was no sign of protein loss in the vaccine bulk. VP2 epitope in both vaccine bulk lots remained relatively stable at 4°C, but there was trend of epitope loss after 1 and 3 months at 25°C storage for Lot #1 and 3, respectively. Based on Western blot analyses using two different monoclonal antibodies N16 specific for the N-terminal peptide of VP1 and MAB979 specific for VP2 shown in Figure 4B and C, there was no EV71 VP1- or VP2-specific protein degradation (no protein fragments with MW below either VP1 and/or VP2 were observed) in the vaccine bulks Lot #1 and 3 stored at 4°C for 12 months. Mouse immunogenicity studies with both vaccine bulks stored at 4°C for 0, 1, 2 and 3 months (2×2 µg/dose immunization at 2 weeks apart) revealed that these samples were highly immunogenic and elicited similar geometric mean titer (GMT) of virus neutralization against E59 vaccine strain ranging from 200 to 600 (data not shown). No contamination was observed in all sterility tests. Together, these stability profiles indicate that the EV71 vaccine bulks produced from the current manufacturing process stored at 4°C for 1 year are sterile, stable, and immunogenic in mice.

**Table 3:** Stability profiles of EV71 vaccine bulks.

	Vaccine Bulk Lot #1 stored at different temperature				Vaccine bulk Lot #3 stored at different temperature			
Time	Total protein (mcg/mL)		VP2 epitope (Unit/mL)		Total protein (mcg/mL)		VP2 epitope (Unit/mL)	
	4°C	25°C	4°C	25°C	4°C	25°C	4°C	25°C
Initial	57.01	57.01	198.35	227.54	48.76	48.76	199.80	199.80
2 weeks	ND	60.73	ND	181.65	ND	50.86	ND	209.55
1 month	58.75	58.25	198.29	150.59	47.74	48.46	206.02	191.36
3 month	58.55	60.50	168.69	156.76	52.59	51.31	192.48	170.48
6 month	57.70	59.27	188.05	137.49	52.19	53.76	190.29	168.52
9 month	58.75	ND	180.35	ND	51.54	51.07	218.64	145.36
12 month	47.76	ND	174.03	ND	46.18	ND	214.39	ND

The total protein and VP2 antigen content in the vaccine bulks were determined by BCA method and VP2 Q-ELISA, respectively.

The vaccine products were analyzed using a Hitachi H-7650 electron microscope according to previously reported protocols [10], some free inactivated EV71 particles were observed and not absorbed onto the alum (data not shown). When the kinetics of vaccine absorption to alum were examined, >50% of protein in the EV71 vaccine product was absorbed within 1 hour after mixing the vaccine bulk with the aluminum phosphate at room temperature. The current data from 3 and 6 months storage also confirm that the rate of alum absorption is more efficient at 25°C in both Lot #1 and 3 (Table 4). In addition, it seems that the virus neutralization titers elicited in mice immunized with the EV71 vaccine product were correlated with the percentage of protein absorbed to alum, particularly in Lot #3 (913 vs. 400 at 4°C and 850 vs. 335 at 25°C). The stability profile of the EV71 vaccine product is supported by no obvious losses of IgG and virus neutralization titers in mouse immunogenicity studies. In the current 18-month stability program, the mouse immune responses also reveal that the EV71 vaccine products from both lots are highly stable for long term storage at 4°C. Finally, no contamination was observed in sterility tests performed at 0, 3, 12 and 18 months.

**Table 4:** Stability profiles of EV71 vaccine products (70 µg of formalin-inactivated EV71 virion formulated with 9 mg of aluminum phosphate in 3 mL of PBS.

Time		Lot #1			Lot #3		
		Unabsorbed Protein (µg/mL)	IgG (GMT ± SE)	Neutralization Titer (GMT ± SE)	Unabsorbed Protein (µg/mL)	IgG (GMT ± SE)	Neutralization Titer (GMT ± SE)
Initial		23.3	4525±705	443±279	23.3	2934±705	233±99
1 month		6.57±0.16	1037±316	153±52	8.56±0.30	1131±176	265±27
2 month		7.69±0.35	2075±353	105±89	9.77±0.04	1903±262	246±14
3 month	4°C	7.15±2.58	1600±330	120±58	9.34±1.19	2263±302	400±88
	25°C	5.00±0.01	2075±353	269±110	6.46±0.12	3200±761	913±125
6 month	4°C	8.25±0.22	1345±280	79±29	9.88±0.11	2691±262	335±83
	25°C	5.36±0.23	1745±300	262±122	7.07±0.37	3200±659	850±122
9 month		5.59±0.08	2986±413	534±90	8.00±0.19	3200±0	1089±187
12 month		5.87±0.06	4222±523	301±86	8.00±0.20	3676±427	349±85
18 month		7.03±0.51	1393±227	534±77	8.82±0.83	2599±244	643±97

Eight mice per group were immunized twice with 0.2 mL of EV71 vaccine products Lot #1 and 3 that were stored in stability study chambers with different pre-set temperatures. The immunization protocol, IgG tier and virus neutralization assay are described in the Materials and Methods section.

### Conclusion

Based the current results, the pilot scale manufacturing process of cell-based EV71 vaccine has been successfully developed, optimized, characterized, and quantified. In the up-stream process development, VP-SFM serum-free medium was identified to be the optimal medium for Vero cell growth and EV71 virus production. Gel filtration chromatography was found to be the most cost-effective for downstream purification, resulting in overall virus yields as high as 57% (Table 1). The formalin-inactivated virion vaccine candidate was found to be stable >18 months at 4°C. At microgram-levels, viral proteins formulated with alum adjuvant could induce strong virus-neutralizing antibody responses in mice, rats, rabbits, and non-human primates. These results provide valuable information supporting the current EV71 vaccine candidate going into human Phase 1 clinical trials.
